# Impact of COVID-19 infection among myasthenia gravis patients- a Cerner Real-World Data^TM^ study

**DOI:** 10.1186/s12883-022-02564-x

**Published:** 2022-01-27

**Authors:** Lakshmi Prasanna Digala, Shivika Prasanna, Praveen Rao, Adnan I. Qureshi, Raghav Govindarajan

**Affiliations:** 1 Department of Neurology, University of Missouri Health Care, Columbia, MO 65201 USA; 2 Center for Biomedical Informatics, Department of Electrical Engineering & Computer Science, University of Missouri, Columbia, MO 65201 USA; 3 Department of Health Management & Informatics, University of Missouri, Columbia, MO 65201 USA; 4 Zeenat Qureshi Stroke Institute, St Cloud, MN 56303 USA

## Abstract

**Background:**

Myasthenia gravis (MG) is an auto-immune disease, and the mainstay of therapy is immunomodulation. Such patients are at high risk of acquiring any infections. Hence, we sought to determine the impact of the current global pandemic COVID-19 infection in MG patients.

**Methods:**

For our study, we used Cerner Real-World Data^TM^ that was provided through Cerner’s HealtheDataLab research tool. We ran a database query from January 2019 to July 2020 in our study and identified myasthenia patients with and without COVID-19 infection. To extract these patients’ data, we used ICD 9-CM, ICD-10, and SNOMED-CT codes. We reported the data using means, range, and prevalence rates, and the p-values were calculated using the two-sample t-test and Pearson’s chi-squared test.

**Results:**

In the COVID-19 data set, a total of twenty-seven myasthenia patients were identified with a positive COVID-19 infection, and four were diagnosed with an exacerbation. The male to female ratio was equal and one unknown gender (3.7%) with a mean (± SD) age of 64.33 ± 18.42 years. This study group was compared with a non-COVID-19 data set in which a total of sixty-four myasthenia patients were identified, and twenty-three had an exacerbation. Among the 13 hospitalized patients in the two groups, the mean length of hospitalization for the myasthenia patients in the COVID-19 data set was 8.28 days (*n* = 7), and the non-COVID-19 set was 4.33 days (n = 6), and it was statistically significant (p-value= 0.007).

**Conclusions:**

The mean length of hospital stay is prolonged in myasthenia patients who tested positive for COVID-19.

## Background

Myasthenia Gravis is an auto-immune disease characterized by fluctuating weakness in the voluntary muscles caused by antibodies towards the neuromuscular junction. When severe weakness develops in the respiratory muscles, it leads to a significant life-threatening complication called myasthenic crisis, resulting in respiratory failure [1]. Around 15 to 20% of myasthenic patients develop exacerbation that requires intubation resulting in hospitalization once in a lifetime [2, 3]. The mainstay of therapy is immunomodulation, and myasthenia patients are at high risk of acquiring any infections. The current global pandemic caused by the COVID-19 predominantly presents with fever, tachypnea, and dyspnea, causing hypoxemic respiratory failure in severe cases [4]. Hence, we sought to determine the impact of [Coronavirus] COVID-19 infection in these patients.

## Materials and methods

For our study, we used Cerner Real-World Data^TM^ provided through Cerner’s HealtheDataLab research tool [5]. This is a retrospective, observational study utilizing real-world data. The COVID-19 dataset in HealtheDataLab contained de-identified patient data of one hundred and seventeen thousand (117 K) patients from 62 contributing health systems after a database refresh in July 2020. The dataset contained all patients tested for COVID-19 at some point during their visits to one of the 62 health centers. The database contained tables with names like *condition, demographics, COVID-19 labs, encounters*, and *medication* that contained information for each of the de-identified patients. Note that the database undergoes a frequent refresh to keep the patients’ data up to date.

To begin with, all patients that had myasthenia gravis were identified from the *condition* table using the ICD-9-CM codes (i.e., 358.0 and 358.01), ICD-10-CM codes (i.e., G70.00 and G70.01), and SNOMED-CT codes (i.e., 91,637,004, 230,686,005, 193,207,007, 230,685,009, 77,461,000,119,109, 77,471,000,119,109, 31,839,002, 55,051,001, 80,976,008) irrespective of their COVID-19 test result. We chose an exhaustive list of codes to avoid missing any myasthenia gravis patients in the database. The database revealed a total of 91 patients that had myasthenia gravis. A SQL join was done on the *condition* and *COVID labs* table to extract myasthenia gravis patients that had received COVID-19 testing along with the test date. The patients were then divided into two sets: one with a positive COVID-19 test and the other set that contained patients that always tested negative for COVID-19. To find patients who always tested negative for COVID-19, we computed the set difference between all patients with myasthenia gravis (91) and those patients who had this condition and tested positive for the COVID-19 (27), and then manually verified the result. This set of non-COVID-19 patients contained 64 patients. For each patient in both the sets, we extracted information such as race, ethnicity, and gender from the *demographics* table. We extracted information on patient complications, comorbidities, and first-reported-date-of-condition from the *condition* table. We also extracted start date and end date of medications that were prescribed after the COVID-19 test result from the *medication* table. Finally, we extracted the discharge disposition and length of stay after the COVID-19 test result from *encounter* table. The number of deceased patients were obtained from the *demographics* table and were then verified using the latest discharge disposition from the *encounter* table. We report the data using means, range, prevalence rates in these two sets of patients. The p-values were calculated using the two-sample t-test and Pearson’s chi-squared test. The p-values are also reported for statistical significance (<0.05).

## Results

Twenty-seven myasthenia patients were identified with a positive COVID-19 infection with a mean (± SD) age of 64.33 ± 18.42 years. In the non-COVID myasthenia study group, sixty-four patients were identified, with a mean (± SD) age of 63.23 ± 18.60 years. The male-to-female ratio, mean age, race, and medications are described in Table 1, and the comorbidities and complications and their p-values in Table 2.

**Table 1:** Mean age, gender, race, and myasthenia drug distribution in the two groups

	COVID Patients with myasthenia (*n* = 27)		Non-COVID Patients with myasthenia (*n* = 64)	
Mean age (±SD)	64.33 ± 18.42 (*n* = 27)		63.23 ± 18.60 (*n* = 64)	
Mean age (±SD) for patients with exacerbation	51.5 ± 14.93 (*n* = 4)		64.13 ± 17.95 (*n* = 23)	
Female	13	48.15%	36	56.25%
Male	13	48.15%	27	42.19%
Unknown	1	3.70%	1	1.56%
Caucasian	16	59.26%	46	71.88%
African American	4	14.81%	8	12.50%
Another racial group	7	25.93%	10	15.62%
Immune globulin intravenous	7	25.93%	13	20.31%
Methylprednisolone	9	33.33%	21	32.81%
Prednisone	18	66.67%	37	57.81%
Mycophenolate Mofetil	6	22.22%	3	4.69%
Pyridostigmine	16	59.26%	33	51.56%

**Table 2:** Comorbidities and complications in COVID-19 positive and non-COVID patients with myasthenia

**Co-morbidities**	**COVID Patients with myasthenia (n= 27)**		**Non-COVID Patients with myasthenia (n=64)**		**P-value**
Hypertension	1	3.70%	4	6.25%	0.986
Diabetes mellitus	5	18.52%	6	9.38%	0.384
Obstructive Sleep apnea	5	18.52%	12	18.75%	0.979
Hyperlipidemia	9	33.33%	32	50.00%	0.144
Atrial fibrillation	3	11.11%	13	20.31%	0.452
Obesity	0	0.00%	1	1.56%	0.654
COPD	4	3.70%	13	4.69%	0.538
**Complications**	**COVID Patients with myasthenia (n= 27)**		**Non-COVID Patients with myasthenia (n=64)**		**P-value**
Pneumonia	13	48.15%	28	43.75%	0.7
Septic shock	9	33.33%	13	20.31%	0.185
Acute Respiratory failure	10	37.04%	22	34.38%	0.808
Chronic Respiratory failure	1	3.70%	3	4.69%	0.725

*Abbreviation: COPD *Chronic Obstructive Pulmonary Disease

Among the COVID-19 positive myasthenia patients, thirteen developed pneumonia, nine patients developed septic shock, and ten patients developed acute respiratory failure. Out of four patients diagnosed with exacerbation, two expired, one got discharged home, and the other was discharged to skilled nursing facility. Among the 13 hospitalized patients in the two groups, the mean length of hospitalization for the myasthenia patients in the COVID-19 data set was 8.28 days (n = 7), and the non-COVID-19 data set was 4.33 days (n = 6), and it was statistically significant (p-value= 0.007).

## Discussion

To determine the impact of COVID-19 on myasthenia patients, so far [CARE-MG] COVID-19 Associated Risks and Effects in Myasthenia Gravis, a physician-reported registry is currently open and active [6]. In addition, two case reports, a single case series, and one retrospective study on Myasthenia patients with COVID-19 infection were reported. One case describes the myasthenic crisis secondary to COVID-19 infection [7]. Another case reported is a myasthenia patient with positive COVID-19 infection with a successful outcome [8]. In a case series, five myasthenia patients with positive COVID-19 were reported [9]. A retrospective study in Brazil was recently published describing the characteristics and outcomes of the COVID-19 positive Myasthenia patients [10]. The patients’ outcomes reported in the cases and studies are highly variable.

Our study utilized the de-identified real-world data from the CERNER database, and the data was analyzed to report the characteristics of the patients and the outcomes. Comorbidities like hypertension, diabetes mellitus, cardiovascular disease, and respiratory diseases may pose a significant risk of developing complications in patients with COVID-19 infection [11, 12]. In our study, although we observed the complications in both COVID and non-COVID groups, no statistically significant difference was observed based on the computed p-values.

Infections are the most common triggering factors to develop exacerbation in myasthenia patients [13, 14]. Acute myasthenia exacerbation can present in different forms, for example, dysphagia, acute respiratory failure, or significant functional disability precluding physical activity. The causes can be different, including inadequate treatment, procedure-related, medical noncompliance, medication to be avoided, and diagnostic errors. Factors potentially involved in myasthenia exacerbation include late-onset MG (above 50 years) and the presence of other autoimmune diseases [14, 15]. In our study, we could not retrieve the characteristics/factors of the patients in detail that might have predisposed them to develop an episode of exacerbation besides COVID-19 infection. We identify it as a limitation to our study.

A single case of myasthenic crisis secondary to COVID-19 infection was reported [9]. The combined use of Hydroxychloroquine and Azithromycin might have precipitated the crisis in that patient [9]. In our study, four patients with COVID-19 infection developed exacerbation, and the precipitating factor is likely to be the COVID-19 infection.

Among the 27 COVID-19 positive myasthenia patients, seven patients were hospitalized, and the calculated mean length of stay is 8.28 days. The mean length of hospitalization in the non-COVID-19 group was 4.33 days. Only 6 out of 64 myasthenia patients were hospitalized in the non-COVID-19 group. Our study observed *a statistically significant* higher mean length of hospitalization of 8.28 days in the COVID-19 group among the hospitalized patients as compared to 4.33 days in the non-COVID-19 group. In an observational study, the average length of hospitalization from the myasthenia exacerbation was reported as 6.5 days [14]. In a large national registry study in Finland, the hospital’s median length of stay with an exacerbation reported was around six days secondary to infection [16].

The mean length of hospital stay is prolonged in myasthenia patients who tested positive for COVID-19.

The study protocols were approved by the Institutional review of Board committee at University of Missouri Columbia. All methods were carried out in accordance with relevant guidelines and regulations of the committee. The ethic committee waived the informed consent as it is de-identified data. The limitations of our study are that we lack the availability of the data publicly. However, in all the centers located in the US, we lack demographical localization of the health centers and lack of seropositivity results in all myasthenia patients.

## Data Availability

The data that support the findings of this study are available from the CERNER, but restrictions apply to the availability of these data, which were used under license for the current study, and so are not publicly available.
